# Synthesis and properties of quinazoline-based versatile exciplex-forming compounds

**DOI:** 10.3762/bjoc.16.101

**Published:** 2020-05-28

**Authors:** Rasa Keruckiene, Simona Vekteryte, Ervinas Urbonas, Matas Guzauskas, Eigirdas Skuodis, Dmytro Volyniuk, Juozas V Grazulevicius

**Affiliations:** 1 Department of Polymer Chemistry and Technology, Kaunas University of Technology, K. Baršausko g. 59, Kaunas 51423, Lithuania

**Keywords:** carbazole, dimethyldihydroacridine, exciplex, phenothiazine, quinazoline

## Abstract

Three compounds, bearing a quinazoline unit as the acceptor core and carbazole, dimethyldihydroacridine, or phenothiazine donor moieties, were designed and synthesized in two steps including a facile copper-catalyzed cyclization and a nucleophilic aromatic substitution reaction. The photophysical properties of the compounds, based on theoretical calculations and experimental measurements, as well as the electrochemical and thermal properties, are discussed. The synthesized compounds form glasses with glass-transition temperatures ranging from 116 °C to 123 °C. The ionization potentials estimated by cyclic voltammetry of the derivatives were in the range of 5.22–5.87 eV. The 3,6-di-*tert*-butylcarbazole-substituted quinazoline-based compound forms a sky-blue emitting exciplex in solid mixture with the acceptor 2,4,6-tris[3-(diphenylphosphinyl)phenyl]-1,3,5-triazine as well as an orange emitting exciplex with the donor 4,4′,4″-tris[3-methylphenyl(phenyl)amino]triphenylamine. A white OLED based on these versatile exciplex systems with a relatively high maximum brightness of 3030 cd/m^2^ and an external quantum efficiency of 0.5% was fabricated.

## Introduction

Organic luminescent materials are extensively used in a wide range of optoelectronic devices. For the design of compounds, potentially exhibiting properties such as bipolar charge transport, delayed fluorescence or aggregation-induced emission enhancement (AIEE), a useful strategy is to employ both donor and acceptor moieties in a single molecular structure [1–4].

Quinazoline is a planar aromatic heterocyclic compound with the fused bicyclic structure consisting of benzene and pyrimidine rings. Quinazoline derivatives were investigated and used in medicinal applications, such as monitoring of specific biological activities and as antimalarial and anticancer agents [5–6]. However, electroactive properties of derivatives of this acceptor have been scarcely reported. Quinazoline-based compounds were used as hosts for red phosphorescent OLEDs with an external quantum efficiency (EQE) of 19.2% [7]. Two blue emitters based on fluorene-bridged quinazoline and quinoxaline derivatives were used in the active layers of OLEDs with EQEs of 1.58% and 1.30%, suggesting that the self-aggregation of emitters had a considerable effect on the photoluminescent and electroluminescent properties [8]. A quinazoline-based emitter exhibiting thermally activated delayed fluorescence (TADF) was also reported [6] and green to yellow TADF OLEDs were fabricated with EQEs from 17.6 to 20.5%. The multicolor emission of a quinazoline–carbazole compound was employed in white OLEDs. White photoluminescence and electroluminescence based on blue emissive quinazoline derivatives obtained through controlled acid protonation were employed in a single-layered white OLED with EQEs of 1.4% and 3% [9]. These reports proved that by using an asymmetric quinazoline acceptor, highly efficient TADF materials for OLEDs could be obtained with easy modulation of the electroluminescent properties. Recently, the first examples of versatile exciplex-forming materials which can form two different types of exciplexes, donor–acceptor/donor and acceptor/donor–acceptor, were reported for simplified non-doped white OLEDs [10]. However, the maximum external quantum efficiencies (EQEs) of such white exciplex-based OLEDs did not exceed 3.2%. It is predictable, that investigation of such versatile exciplex-forming materials will allow to develop highly efficient exciplex-based OLEDs.

With the above mentioned aim, herein we report on the synthesis and properties of electroactive compounds bearing a quinazoline moiety as an acceptor and the widely used carbazole, phenothiazine and dimethyldihydroacridine species as donors [11–13].

## Results and Discussion

### Synthesis and thermal properties

**Scheme 1:**
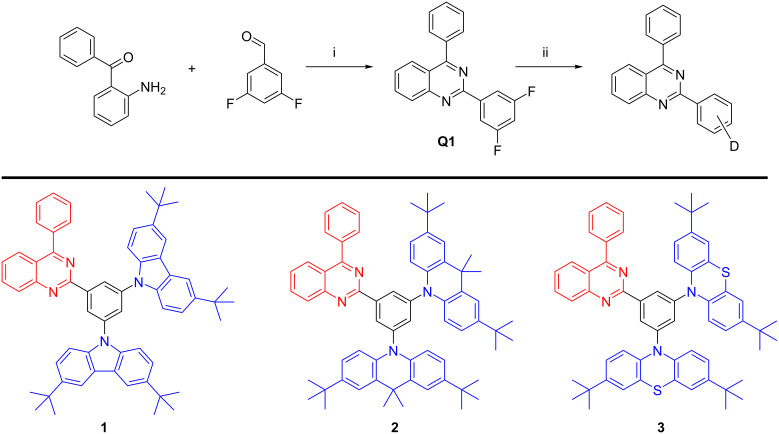
Synthesis of quinazoline derivatives **1–3**. Conditions: i) ammonium acetate, copper(II) chloride, isopropanol, reflux, 24 h; ii) donor moiety (D), NaH, DMF, reflux, 24 h.

Aromatic rigid moieties were selected in the build-up of the new electroactive compounds. The commonly used carbazole, phenothiazine, and dimethyldihydroacridine donor units and the scarcely used quinazoline unit as the electron acceptor were chosen for the design of the compounds (Scheme 1).

A facile and reliable one-pot three-component method was used for the quinazoline formation by refluxing 2-aminobenzophenone, difluorobenzaldehyde, ammonium acetate, and CuCl_2_ in ethanol. The synthetic method using a cheap catalyst, easy workup, and the high yield (78%) of quinazoline **Q1** makes the compound a promising candidate as an electron acceptor in donor–acceptor systems [14]. The target compounds were obtained by nucleophilic substitution reaction of the intermediate quinazoline derivative **Q1** with the corresponding donor compounds. The chemical structures were characterized by ^1^H NMR, ^13^C NMR, ATRIR spectroscopy and mass spectrometry.

**Figure 1:**
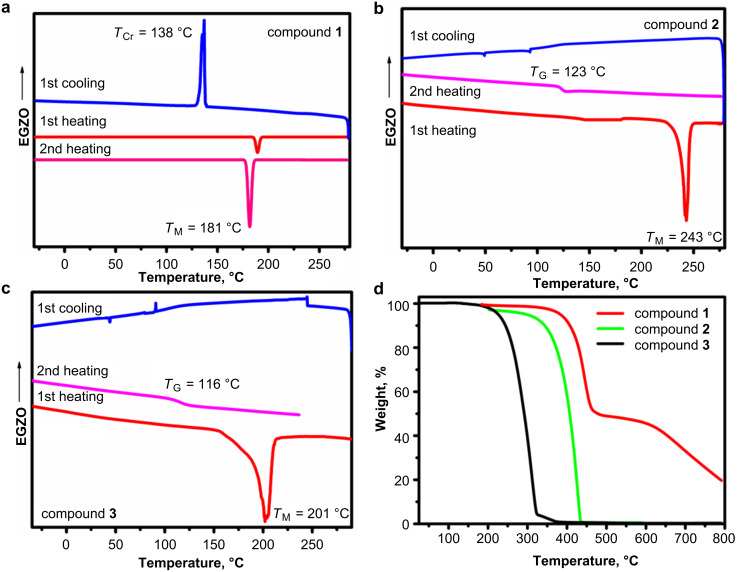
DSC (a, b, c) and TGA (d) curves of compounds **1–3**. Scan rates were 20 °C/min (TGA) and 10 °C/min (DSC).

All compounds **1**–**3** were obtained as crystalline substances. The thermal characteristics were determined by differential scanning calorimetry (DSC) and thermogravimetric analysis (TGA). During the first DSC heating scan (Figure 1a–c), melting signals of compounds **1**–**3** were detected in the range from 181 °C to 243 °C. Compounds **2** and **3**, bearing dimethyldihydroacridine and phenothiazine moieties formed molecular glasses. The glass transitions were detected during the cooling and the second heating scans at 123 °C and 116 °C, respectively. No glass transition was detected for compound **1**, that only showed a crystallization signal (*T*_CR_ = 138 °C) during the cooling scan. The 10% weight-loss temperature (*T*_D−10%_) of the quinazoline-based compounds decreased in the order of **3** (409 °C) > **2** (345 °C) > **1** (247 °C). For compounds **1** and **2** a complete weight loss was observed in the TGA experiments. This observation showed, that the weight loss of these compounds apparently was due to sublimation. The carbon residue of compound **3** was attributed to the tendency of the phenothiazine moiety to decompose rather than to sublimate, as it has been observed for similar phenothiazine-based compounds [15].

### Theoretical calculations and electrochemical properties

**Figure 2:**
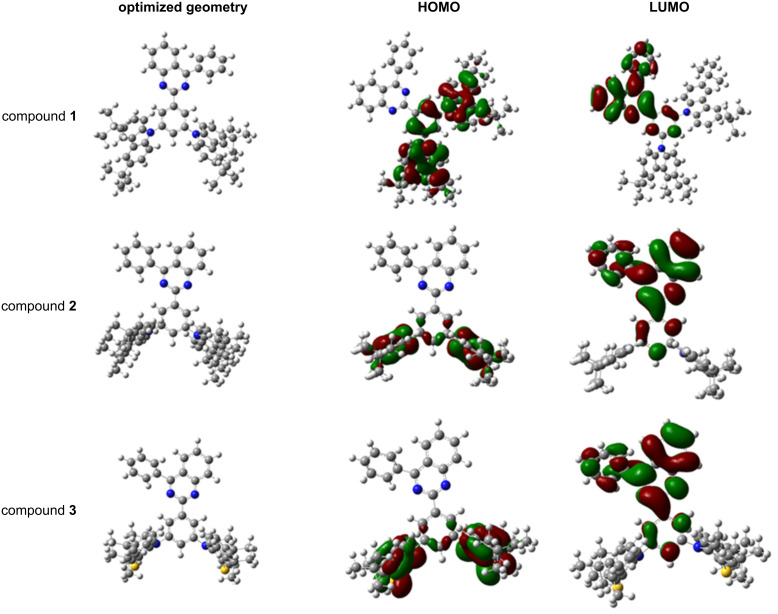
Frontier-orbital distributions and optimized geometries at the ground state of quinazoline-based compounds **1**–**3**, calculated at the B3LYP/6-31G (d, p) level of theory.

DFT calculations were employed to gain insight into the structure–property relationships of the quinazoline-based derivatives **1–3**. The compounds have phenyl spacers between the donor and acceptor units (Figure 2). Therefore, the dihedral angle values were estimated between the bonded aromatic unit and the respective substituents. All compounds showed large twisting angles that indicated HOMO and LUMO separation and a controlled conjugation distance. In the optimized ground-state geometries, the acceptor and the phenyl units are plane, whereas the dihedral angles between the donor moieties and phenyl unit varied and were found to be 126° for **1**, 90° for **2**, and 118° for **3**.

The twisted configurations of compounds **2** and **3** fully separated the HOMO and LUMO orbitals. The oscillator strengths were 0.0 indicating that it is spin-forbidden due to a quasi-orthogonal geometry of the donor part on the acceptor core, and the charge-transfer (CT) character [16].

On the other hand, not adapting the vertical dihedral angle enabled a slight HOMO and LUMO overlap in the geometry of compound **1**. This resulted in a higher oscillator strength of 0.0109. As shown in Figure 2, the LUMOs were located on the central electron-accepting quinazoline core and the phenyl unit, whereas the HOMOs were mainly localized on the electron-donating peripheral substituents.

**Figure 3:**
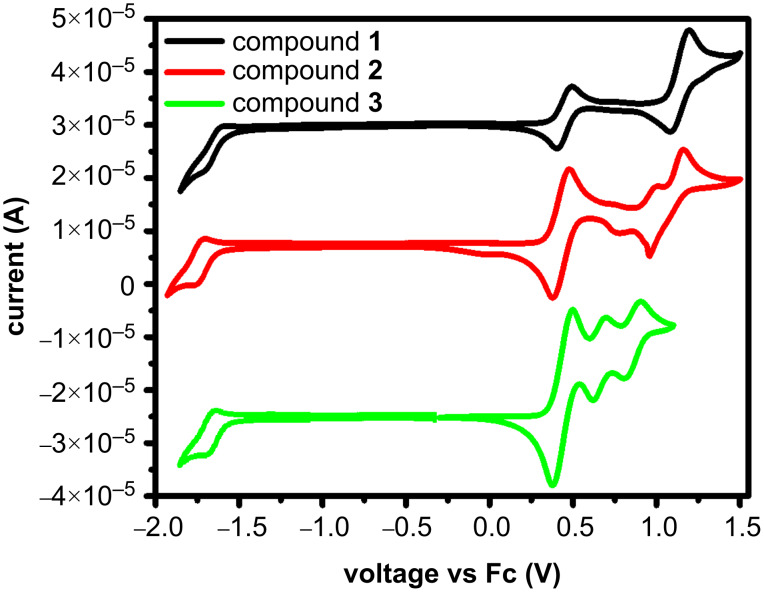
Cyclic voltammograms of quinazoline-based compounds **1**–**3**.

The HOMO energy values (Table 1) showed dependence on the donating characteristics of the substituents of the quinazoline derivatives **1**–**3**, whereas the LUMO energies were close and characteristic of the quinazoline unit. The electron-donating characteristics were investigated experimentally by cyclic voltammetry (CV). All three compounds showed reversible oxidation (Figure 3) and the data are summarized in Table 1.

The ionization potential (IP_CV_) and electron affinity (EA_CV_) values were estimated accordingly from the oxidation and reduction onset potentials against ferrocene (*E*_ox_/*E*_red,onset vs Fc_). The IP_CV_ values demonstrated the similar and collaborative electron-donating effect of the carbazole, phenothiazine, and 2,7-di-*tert*-butyl-9,9-dimethylacridine donor moieties on the electron-releasing energy.

**Table 1:** Electrochemical characteristics.

Compound	*E*_red. vs Fc_^a^, V	*E*_ox. vs Fc_^b^, V	IP_CV_^c^, eV	EA_CV_^d^, eV	*E*_g_^CVe^, eV	HOMO^f^, eV	LUMO^g^, eV

**1**	−1.70	0.77	5.87	3.39	2.48	5.13	2.10
**2**	−1.59	0.31	5.41	3.51	1.90	4.64	2.10
**3**	−1.59	0.12	5.22	3.51	1.71	4.74	2.18

^a^Onset reduction potential of the sample vs onset oxidation potential of ferrocene; ^b^onset oxidation potential of the sample vs onset oxidation potential of ferrocene; ^c^ionization potential, IP_CV_ = *E* onset oxidation vs Fc +5.1 eV [17–18]; ^d^electron affinity, EA_CV_ = 5.1 eV − *E*_red_ vs Fc; ^e^electrochemical bandgap *E*_g_^CV^ = IP_CV_ − EA_CV_; ^f^theoretically calculated HOMO energy; ^g^theoretically calculated LUMO energy.

The phenothiazine-substituted quinazoline compound **3** required the lowest energy for electron release compared to the other compounds studied. The electron affinity values were comparable as they characterize the electron-withdrawing abilities of the quinazoline moiety present in all compounds. The energy bandgap values estimated from CV measurements indicated a more extended π-electron conjugation system for compound **1** compared to compounds **2** and **3**. These results correlated well with the results of theoretical calculations which revealed that the HOMO and LUMO orbitals of the carbazole-substituted quinazoline compound **1** overlap.

### Photophysical and electronic properties

**Figure 4:**
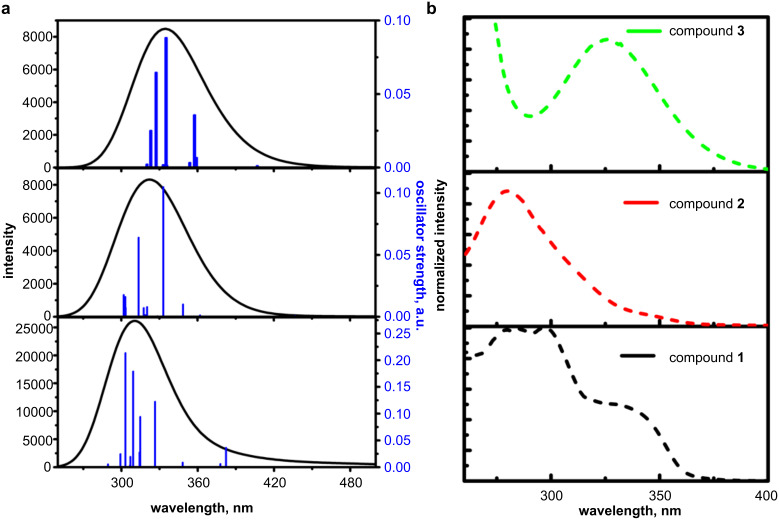
UV–vis absorption spectra of compounds **1**–**3**. a) Theoretical and b) experimental spectra of compounds **1**–**3** in THF solution.

Figure 4 shows the theoretical UV spectra and experimental absorption spectra of dilute THF solutions of compounds **1**–**3**. The theoretical UV–vis spectra of the derivatives had single absorption bands. The band at ca. 310 nm was characterized by a combination of various transitions towards several excited states. The theoretical UV spectrum of derivative **1** had a shoulder at 390 nm. In general, the experimental UV spectra of the derivatives **1**–**3** were consistent with the theoretical ones. The UV spectra of compounds **1**, **2**, and **3** had lowest energy bands (LEB) at 350, 280, and 325 nm, which were the main characteristics of the donor moieties in the structures, i.e., the carbazole, dimethyldihydroacridine, and phenothiazine moieties, respectively [19]. Additionally, a weak absorption band (shoulder) at ca. 348 nm was detected for compound **2**, evidencing an intramolecular charge transfer (ICT) between the phenothiazine and quinazoline units. The energy band gap values determined from the edges of the experimental UV spectra of the derivatives **1**–**3** correlated with electrochemically estimated ones and with the ionization potential values discussed above. The photophysical characteristics determined from the UV–vis absorption and photoluminescence spectra are collected in Table 2.

**Figure 5:**
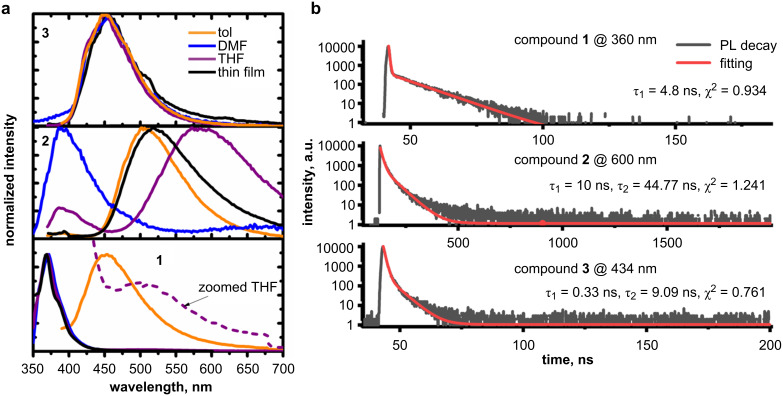
Fluorescence spectra (a) of dilute solutions and thin films of compounds **1**–**3** (λ_exc_ = 350 nm and PL decay curves (b) of thin films of derivatives **1**–**3** recorded at different emission wavelengths.

The emission spectra of diluted toluene solutions of derivatives **1** and **2** displayed broad Gaussian forms, whereas the emission spectrum of compound **3** was narrow and slightly vibrational (Figure 5). The strongly red-shifted fluorescence bands (ca. 107 nm for **1** and 156 nm for **2**) with respect to the LEB of absorption, were attributed to charge-transfer (CT) transitions [20]. The PLQY quantum yields of the dilute solutions did not exceed 2%.

**Table 2:** Photophysical properties of compound **1**–**3**.

Compound	λ_abs_^a^, nm	λ_PL_^b^, nm	PLQY, %	*E*_g_^opt c^, eV

medium	THF	toluene solution (thin film)		

**1**	301, 331, 345	453 (370)	1 (6)	3.47
**2**	280, 348	506 (521)	2 (5)	3.39
**3**	325	450 (450)	1 (2)	3.29

^a^λ_abs_ are wavelengths of absorption maxima; ^b^λ_PL_ are wavelengths of emission maxima; ^c^E_g_^opt^ is optical band gap estimated as 1240/λ_abs_ onset where λ_abs_ onset is the wavelength of the onset of absorption.

The dependence of the emission wavelengths of the derivatives on the solvent polarity was also tested. Figure 5 shows the emission spectra of the compounds dissolved in three solvents with increasing polarities, i.e., toluene (0.099), tetrahydrofuran (0.207) and dimethylformamide (0.4) (values in parentheses are the solvent polarities relative to water) [21]. The PL spectra of the solutions of compound **3** appeared to be solvent-polarity independent as only the structured emission band of the locally excited (^1^LE) state at 450 nm was observed. In contrast, compound **2** bearing dimethyldihydroacridine as the donor moiety, possessed the most sensitive ^1^CT. A positive solvatochromism was observed resulting in bathochromic shifts of the PL spectra in the solvents with increasing polarizability indexes. Interestingly, the intensive emission band of the locally excited (^1^LE) state at 400 nm and a weak ^1^CT state at ca. 677 nm were also present in the PL spectrum of the solution of compound **2** in the polar solvent DMF. The PL spectra of the solutions of compound **1** in polar solvents were dominated by the band of the ^1^LE transition at 375 nm. A ^1^CT state could be formed for compound **1** as evidenced by the PL spectrum of its solution in low-polarity toluene. A weak low energy ^1^CT band was also observed for the solution in THF with moderate polarity (see the zoomed spectrum of THF solution of compound **1**, Figure 5a).

**Figure 6:**
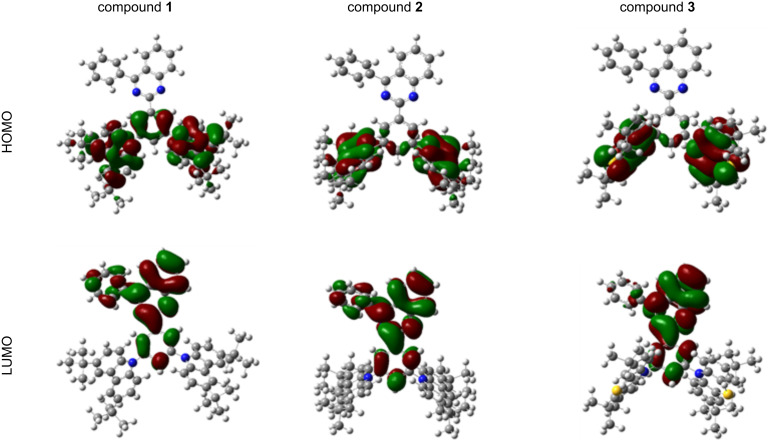
Electron and hole NTOs of compounds **1**–**3** in the S1 excited state (vacuum).

To assist in the analysis of the spectra, natural transition orbitals (NTO) for the S1 state were generated. Figure 6 shows the pairs of electron–hole NTOs for the relaxed S1 excited-state geometry. These orbitals indicate which changes in electronic density occur upon relaxation. For all three compounds, the transition took place between orbitals delocalized over both donor fragments and the quinazoline moiety indicating a charge transfer in vacuum. Considering the experimental data, it could be presumed that this ^1^CT state is very sensitive and easily quenched in polar solvents as it competes with a ^1^LE transition originating from the donor moieties.

The emission spectra of solid samples of compounds **2** and **3** were broad and had ^1^CT character (Figure 5). Meanwhile, PL of the solid sample of compound **1** originated from the ^1^LE state. The PL spectrum of the solid sample was identical to that of the solution in a polar solvent, apparently because of the high polarity of compound **1**. Furthermore, the emission of the solid sample was more efficient than that of the solution, with a PLQY of up to 6%. In order to determine the origin of the emission, PL decay curves of the solid samples of compounds **1**–**3** were recorded (Figure 5b). The PL decay curve of the solid sample of the carbazole-containing compound (**1**) was adequately described by a single-exponential function with a lifetime of 4.8 ns (prompt fluorescence). The PL decay curves of compounds **2** and **3** were found to be double exponential with the lifetimes of both components in the ns range. Thus, compounds **2** and **3** were characterized by prompt fluorescence and no delayed fluorescence was observed. The double-exponential decays were apparently related to the spectral diffusion (exciton migration and localization at lower energy states) which is usually detected for materials exhibiting low-intensity emissions [22–23].

### Exciplex-forming properties

**Figure 7:**
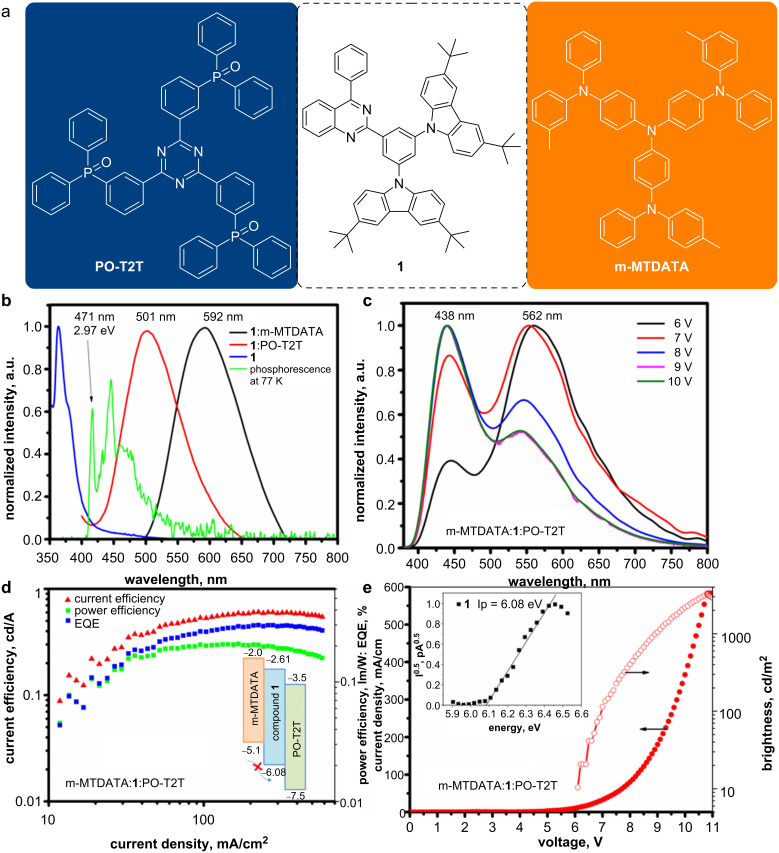
Chemical structures of exciplex-forming materials used, and visualization of white electroluminescence obtained by mixing sky-blue and orange exciplexes (a). PL spectra of the film of **1**, of exciplexes m-MTDATA:**1**/**1**:PO-T2T and phosphorescence spectrum of the solution of compound **1** in THF at 77 K (b). Phosphorescence was recorded with the delay of 50 ms after UV excitation (300 nm). EL spectra of OLED based on m-MTDATA:**1**:PO-T2T recorded at different voltages (c). EQE, current and power efficiencies of OLED and equilibrium energy diagram for the light-emitting layer m-MTDATA:**1**:PO-T2T (d, inset). Current density and brightness versus voltages for the device and photoelectron emission spectrum of the solid sample of compound **1** (e, inset).

Since compound **1** was characterized by the most blue-shifted fluorescence in the solid-state and a high first triplet energy level of 2.97 eV (Figure 5a and Figure 7a), this compound was regarded as a promising candidate for blue exciplex formation in the solid state. Indeed, compound **1** formed a sky-blue emitting exciplex (501 nm) in a solid mixture with the acceptor 2,4,6-tris[3-(diphenylphosphinyl)phenyl]-1,3,5-triazine (PO-T2T). Moreover, an orange exciplex emission with a PL spectrum peaked at the wavelength of 592 nm was observed for the solid mixture with the donor 4,4′,4″-tris[3-methylphenyl(phenyl)amino]triphenylamine (m-MTDATA) (Figure 7b). The combination of the sky-blue and orange exciplex emissions may result in white electroluminescence (EL). To check this assumption, a non-doped OLED with three light-emitting layers comprising m-MTDATA:**1**:PO-T2T was fabricated (Figure 7c). The structure of the device was as follows: HAT-CN (10 nm)/NPB (48 nm)/m-MTDATA (16 nm)/compound **1** (20 nm)/PO-T2T (16 nm)/TSPO1 (4 nm)/TBPi (36 nm). In this device architecture, the common hole/electron injecting/transporting/blocking layers hexaazatriphenylenehexacarbonitrile (HAT-CN), *N*,*N*′-di(1-naphthyl)-*N*,*N*′-diphenyl-(1,1′-biphenyl)-4,4′-diamine (NPB), diphenyl[4-(triphenylsilyl)phenyl]phosphine oxide (TSPO1), 2,2′,2′′-(1,3,5-benzinetriyl)-tris(1-phenyl-1*H*-benzimidazole) (TPBi), and fluorolithium (LiF) were used. Hole–electron recombination was expected at the two interfaces of m-MTDATA:**1** and **1**:PO-T2T, resulting in the formation of orange and sky-blue exciplex emissions, respectively. The EL spectrum of the device was characterized by two bands with maximum wavelengths of 438 and 562 nm. The blue shift of the orange exciplex emission of m-MTDATA:**1** (the intensity maximum in the PL spectrum was detected at 592 nm), could be explained by an overlapping with blue emission. However, the blue shift of the sky-blue emission of the exciplex **1**:PO-T2T could not be explained by the same reason. Most probably, the EL band at 438 nm was related to the emission of m-MTDATA. Indeed, the high energy barrier at the interface m-MTDATA:**1** did not allow a hole reaching the interface **1**:PO-T2T (Figure 7d, inset, marked by a crossed arrow) and the maximum EQE value did not exceed 0.5%. The high turn-on voltage of 6.2 V could be attributed to energy barriers due to the high ionization potential (6.08 eV) of compound **1** (Figure 7e, inset). A relatively high maximum brightness of 3030 cd/m^2^ was achieved.

Taking into account the rare detection of versatile exciplex-forming properties of compound **1** both with a donor and with an acceptor, further investigations are required which could result in a better performance of exciplex-based OLEDs.

## Conclusion

Three quinazoline-based derivatives containing different donor moieties were designed and prepared by a two-step synthesis comprising a facile cyclization and a nucleophilic aromatic substitution reaction. The dimethyldihydroacridine and phenothiazine-containing quinazoline compounds formed molecular glasses with glass-transition temperatures of 123 °C and 116 °C, respectively. The ionization potentials estimated by cyclic voltammetry of the derivatives were found to be in the range of 5.22–5.87 eV. The 3,6-di-*tert*-butylcarbazole-substituted quinazoline derivative formed a sky-blue emitting exciplex with the acceptor 2,4,6-tris[3-(diphenylphosphinyl)phenyl]-1,3,5-triazine as well as an orange emitting exciplex with the donor 4,4′,4″-tris[3-methylphenyl(phenyl)amino]triphenylamine. A white OLED was fabricated based on the versatile exciplex-forming systems with a relatively high maximum brightness of 3030 cd/m^2^ and an EQE of 0.5%.

## Experimental

### Instrumentation

^1^H NMR and ^13^C NMR spectra were recorded with a Bruker Avance III apparatus (400 and 101 MHz). The samples were prepared by dissolving ca. 20 mg of the material in 1 mL of deuterated chloroform (CDCl_3_) or dimethylsulfoxide (DMSO-*d*_6_). ^1^H nuclei were excited by using the frequency of 400 MHz. ^13^C nuclei were excited by using the frequency of 101 MHz. The data are presented as chemical shifts (δ) in ppm (in parentheses: multiplicity, coupling constant, and integration). IR spectra were recorded with a Vertex 70 Bruker spectrometer equipped with an ATR attachment with a diamond crystal over frequencies of 600–3500 cm^−1^ with a resolution of 5 cm^−1^ over 32 scans. The IR spectra were presented as a function of transparency (T) expressed in percent (%) against the wavenumber (v) expressed in cm^−1^. Elemental analysis was performed with an Exeter Analytical CE-440 elemental analyzer. Mass spectra were obtained with a Waters ZQ 2000 mass spectrometer. The introduction of the sample into the ion source occurred by coupling a gas chromatograph and a high-pressure liquid chromatograph. The samples were prepared as dilute solutions of the compounds and were ionized by using electrospray ionization. The mass spectra are presented as an abundance of the ion versus the mass-to-charge ratio (*m/z*). Melting points of the compounds were determined with an Electrothermal MEL-TEMP apparatus. Absorption spectra of dilute solutions (10^−4^–10^−5^ mol/L) and thin films of the synthesized compounds were recorded with a Perkin Elmer Lambda 25 spectrophotometer. Fluorescence and phosphorescence spectra of thin films and dilute solutions (10^−4^–10^−5^ mol/L) of the compounds were recorded at room (295 K) and low (77 K) temperatures with a luminescence spectrometer Edinburgh Instruments FLS980. Photoluminescence quantum yields (PLQY) of the solutions and thin films of the materials were measured using an integrating sphere. Phosphorescence spectra were recorded at 77 K. Differential scanning calorimetry (DSC) measurements were carried out using a TA Instruments Q2000 thermosystem. The samples were examined at a heating/cooling rate of 10 °C/min under a nitrogen atmosphere.

Thermogravimetric analysis (TGA) was performed with a TA Instruments Q50 analyzer. The heating rate was 20 °C/min under nitrogen atmosphere. Cyclic voltammetry measurements were performed by using a glassy carbon working electrode (a disk with the diameter of 2 mm) in a three-electrode cell with an Autolab Type potentiostat–galvanostat. The measurements were carried out for the solutions in dry dichloromethane containing 0.1 M tetrabutylammonium hexafluorophosphate at 25 °C; the scan rate was 50 mV/s while the sample concentration was 10^−3^ M. The potentials were measured against silver as a reference electrode. A platinum wire was used as a counter electrode. The potentials were calibrated against the standard ferrocene/ferrocenium (Fc/Fc^+^) redox system [24].

The ground-state geometries were optimized by using the B3LYP (Becke three parameters hybrid functional with Lee–Yang–Perdew correlation) [25] functional at the 6-31G (d, p) level of theory in vacuum with the Gaussian software [26].

Firstly, the equilibrium conformer search at the ground state was performed by using the MMFF (molecular mechanics force fields) method, and this geometry was used for further optimization. The vertical singlet and triplet energy values were calculated by using the energy values at the corresponding excited-state geometry.

The time-dependent DFT (TD-DFT) calculations were carried out with the Gaussian 16 software package and molecular orbitals were visualized by using Gaussview.

Photoelectron emission spectroscopy measurement was performed according to the procedure reported in literature [27]. OLED fabrication and characterization was carried out according to the procedure reported earlier [28].

### Materials

2-Aminobenzophenone, 3,5-difluorobenzaldehyde, ammonium acetate, phenothiazine, sodium hydride, sodium sulfate, *tert*-butyl chloride, zinc chloride (purchased from Aldrich), 9*H*-carbazole, copper(II) chloride (purchased from Reakhim), and 2,7-di-*tert*-butyl-9,9-dimethyl-9,10-dihydroacridine (purchased from Center for Physical Sciences and Technology) were used as received. Thin-layer chromatography was performed using TLC plates covered with silica gel matrix on aluminum backing (purchased from Aldrich).

**3,6-Di-*tert*-butylcarbazole** and **3,7-di-*tert*-butylphenothiazine** were synthesized according to the procedures reported in literature [29].

**2-(3,5-Difluorophenyl)-4-phenylquinazoline (Q1)** was synthesized according to the procedure reported in literature [14]. A mixture of 2-aminobenzophenone (1.1 g, 5.6 mmol), ammonium acetate (1.3 g, 16.9 mmol), 3,5-difluorobenzaldehyde (0.8 g, 5.6 mmol), and CuCl_2_ (1.5 g, 11.2 mmol) in 10 cm^3^ isopropanol was refluxed for 24 h. The progress of the reaction was periodically monitored by thin-layer chromatography. After completion of the reaction, water was added to the mixture until precipitation appeared. The precipitate was collected by filtration and washed with plenty of water to remove excess CuCl_2_, NH_4_OAc, and reduced copper salt. Compound **Q1** (1.4 g, 78%) was obtained as yellowish crystals. ^1^H NMR (400 MHz, CDCl_3_) δ 8.28–8.22 (m, 2H), 8.16 (d, *J* = 9.1 Hz, 2H), 7.96–7.84 (m, 3H), 7.61 (dd, *J* = 9.5, 6.6 Hz, 4H), 6.94 (tt, *J* = 8.6, 2.4 Hz, 1H); ^13^C NMR (101 MHz, CDCl_3_) δ 165.01, 161.01, 156.01, 149.40, 134.00, 133.90, 133.10, 129.24, 128.8, 128.66, 127.76, 127.4, 127.14, 104.70; ATR-IR (solid state on ATR, cm^−1^): 3098 (Ar C–H), 2904 (Alk C–H), 1559, 1371 (Ar C–N), 1110 (Alk C–F); anal. calcd for C_20_H_12_F_2_N_2_: C, 75.46; H, 3.80; F, 11.94; N, 8.80; found: C, 75.41; H, 3.75; F, 11.99; N, 8.85%; exact mass 318.10 g/mol; MS (*m*/*z*): 319 [M + H]^+^.

### General procedure for the synthesis of compounds **1–3**

The target compounds **1**–**3** were synthesized through nucleophilic substitution reactions between quinazoline derivative **Q1** and the respective donor compound in the presence of sodium hydride in dry dimethylformamide (DMF). The reaction mixtures were refluxed for 24 h. After completion of the reactions, the reaction mixtures were poured into water, extracted with chloroform (3 × 50 mL) and dried over sodium sulfate. The compounds were purified by column chromatography using hexane as eluent and recrystallized from acetone.

**9,9'-(5-(4-Phenylquinazolin-2-yl)-1,3-phenylene)bis(3,6-di-*tert*-butyl-9*H*-carbazole) (1):** Quinazoline derivative (**Q1**, 0.25 g, 0.79 mmol), 3,6-di-*tert*-butylcarbazole (0.48 g, 1.7 mmol) and sodium hydride (0.50 g, 1.6 mmol) in dry dimethylformamide (DMF) were used for the nucleophilic substitution reaction. The title compound was obtained as yellowish crystals in a yield of 0.25 g, 37%; *T*_m_ = 181 °C (DSC); ^1^H NMR (400 MHz, CDCl_3_) δ 8.18 (d, *J* = 7.1 Hz, 2H), 8.09 (t, *J* = 7.5 Hz, 2H), 8.00 (s, 4H), 7.87–7.69 (m, 4H), 7.53 (dd, *J* = 9.5, 6.3 Hz, 3H), 7.38 (d, *J* = 8.5 Hz, 4H), 7.25 (d, *J* = 8.5 Hz, 4H), 6.86 (t, *J* = 8.5 Hz, 1H), 1.37 (s, 36H); ^13^C NMR (101 MHz, CDCl_3_) δ 165.01, 161.01, 156.01, 149.40, 144.90, 142.40, 142.24, 138.05, 133.95, 130.22, 129.24, 128.66, 127.76, 127.14, 123.43, 116.19, 110.00, 77.35, 77.03, 76.72, 34.71, 32.05; ATR-IR (solid state on ATR, cm^−1^): 3061 (Ar C–H), 2969 (Alk C–H),) 1490, 1361, 1262 (Ar C–N), 980, 880 (Alk C–H); anal. calcd for C_60_H_60_N_4_: C, 86.08; H, 7.23; N, 6.69; found: C, 86.03; H, 7.20; N, 6.68%; exact mass 836.48 g/mol; MS (*m*/*z*): 836 [M^+^].

**9,9'-(5-(4-Phenylquinazolin-2-yl)-1,3-phenylene)bis(2,7-di-*tert*-butyl-9,9-dimethyl-9,10-dihydroacridine) (2):** Quinazoline derivative (**Q1**, 0.22 g, 0.69 mmol), 2,7-di-*tert*-butyl-9,9-dimethyl-9,10-dihydroacridine (0.58 g, 2.79 mmol) and sodium hydride (0.50 g, 1.6 mmol) in dry dimethylformamide (DMF) were used for the nucleophilic substitution reaction. The title compound was obtained as yellowish crystals in a yield of 0.10 g, 21%; *T*_m_ = 233 °C; ^1^H NMR (400 MHz, CDCl_3_) δ 8.93 (s, 2H), 8.11 (s, 2H), 7.89-7.84 (s, 4H), 7.56 (s, 4H), 7.48 (d, *J* = 7.6 Hz, 3H), 7.04 (s, 4H), 6.95 (s, 3H), 6.57 (d, *J* = 8.1 Hz, 2H), 1.72 (s, 48H); ^13^C NMR (101 MHz, CDCl_3_) δ 165.01, 161.01, 156.01, 149.40, 144.90, 142.40, 134.00, 133.90, 133.10, 132.50, 129.24, 128.8, 128.66, 127.76, 127.4, 127.14, 122.8, 123.50, 77.35, 77.03, 76.72, 34.71, 32.05, 31.0; ATR-IR (solid state on ATR, cm^−1^): 3055 (Ar C–H), 2972 (Alk C–H),) 1495, 1359, 1262 (Ar C–N), 974, 882 (Alk C–H); anal. calcd for compound C_66_H_72_N_4_: C, 86.04; H, 7.88; N, 6.08; found: C, 85.99; H, 7.91; N, 6.10%; exact mass 920.56 g/mol; MS (*m*/*z*): 922 [M + H]^+^.

**9,9'-(5-(4-Phenylquinazolin-2-yl)-1,3-phenylene)bis(3,7-di-*tert*-butylphenothiazine) (3):** Quinazoline derivative (**Q1**, 0.30 g, 0.9 mmol), 3,7-di-*tert*-butylphenothiazine (0.73 g, 1.2 mmol) and sodium hydride (0.5 g, 1.6 mmol) in dry dimethylformamide (DMF) were used for the nucleophilic substitution reaction. The title compound was obtained as yellowish crystals in a yield of 0.194 g, 23%; *T*_m_ =144 °C; ^1^H NMR (400 MHz, DMSO) δ 8.39 (s, 1H), 7.71 (s, 2H), 7.13 (d, *J* = 2.1 Hz, 3H), 7.02–6.88 (m, 11H), 6.75 (d, *J* = 8.3 Hz, 2H), 6.62 (d, *J* = 8.3 Hz, 2H), 6.02 (d, *J* = 7.5 Hz, 3H), 1.27–1.09 (m, 36H); ^13^C NMR (101 MHz, DMSO) δ 165.01, 161.01, 156.01, 149.40, 144.90, 140.80, 138.05, 133.95, 130.22, 129.24, 128.66, 127.76, 124.50, 122.90, 121.60, 115.51, 115.0, 77.35 (s), 77.35, 77.03, 76.72, 34.71, 32.05; ATR-IR (solid state on ATR, cm^−1^): 3069 (Ar C–H), 2971 (Alk C–H),) 1265 (Ar C–N); anal. calcd for C_60_H_60_N_4_S_2_: C, 79.96; H, 6.71; N, 6.22; S, 7.11; found: C, 79.91; H, 6.66; N, 6.27; S, 7.16%; exact mass 900.43 g/mol; MS (*m*/*z*): 901 [M + H]^+^.
